# Accuracy, Confidence, and Experiential Criteria for Lie Detection Through a Videotaped Interview

**DOI:** 10.3389/fpsyt.2018.00748

**Published:** 2019-01-22

**Authors:** Antonietta Curci, Tiziana Lanciano, Fabiana Battista, Sabrina Guaragno, Raffaella Maria Ribatti

**Affiliations:** Department of Education, Psychology, Communication, University of Bari “Aldo Moro”, Bari, Italy

**Keywords:** lie detection, detection accuracy, confidence, experiential criteria, interview

## Abstract

An individual's ability to discriminate lies from truth is far from accurate, and is poorly related to an individual's confidence in his/her detection. Both law enforcement and non-professional interviewers base their evaluations of truthfulness on experiential criteria, including emotional and expressive features, cognitive complexity, and paraverbal aspects of interviewees' reports. The current experimental study adopted two perspectives of investigation: the first is aimed at assessing the ability of naïve judges to detect lies/truth by watching a videotaped interview; the second takes into account the interviewee's detectability as a liar or as telling the truth by a sample of judges. Additionally, this study is intended to evaluate the criteria adopted to support lie/truth detection and relate them with accuracy and confidence of detection. Results showed that judges' detection ability was moderately accurate and associated with a moderate individual sense of confidence, with a slightly better accuracy for truth detection than for lie detection. Detection accuracy appeared to be negatively associated with detection confidence when the interviewee was a liar, and positively associated when the interviewee was a truth-teller. Furthermore, judges were found to support lie detection through criteria concerning emotional features, and to sustain truth detection by taking into account the cognitive complexity and the paucity of expressive manifestations related with the interviewee's report. The present findings have implications for the judicial decision on witnesses' credibility.

## Introduction

One of the main challenges in police investigation and legal proceedings is to assess whether an interviewed suspect, defendant or witness is offering a deceitful account of relevant facts. To corroborate an interviewee's claims, police, and jurors might rely on extrinsic sources of evidence, such as documents, phone tapping, CCTV, GPS-tracked movements, etc. When such external sources are not available—such as in cases of family abuses and maltreatments—interviewers can only focus on the intrinsic qualities of interviews and derive from these qualities some experiential criteria to detect lies.

Classical studies on lie detection have demonstrated that the ability of laypeople to discriminate lies from truth “*is only slightly better than flipping a coin*” [(1), p. 284]: DePaulo et al. (2) combined the results of more than 1,300 estimates of the relationship between behaviors and deceit to identify behavioral cues of deceit. The authors concluded that simply relying on non-verbal behavior to discriminate truth from lies is insufficient, and further evidence is needed to definitively establish if someone is lying or not. In their comprehensive meta-analysis on deception detection accuracy, Bond and DePaulo (3) synthesized the results from 206 documents and 24,483 judges and found that people achieve an average of 54% correct lie-truth discrimination, correctly classifying 47% of lies as deceptive, and 61% of truths as non-deceptive. This proportion only increases marginally for professional lie-catchers: Vrij (4) reviewed studies on deception detection accounting for an accuracy rate of 55.91% for law enforcement personnel, although professionals' evaluation might be biased by overconfidence in their judgements (5). Finally, a meta-analysis by DePaulo et al. (6) yielded a correlation of 0.04 between accuracy ratings and confidence in lie detection, indicating that, even when people feel overconfident in their evaluation, there is no guarantee of detection accuracy.

## Legal Criteria for the Evaluation of Witness Reports: Examples Across Jurisdictions

In spite of scientific evidence, the legal system is forced to identify some reliable criteria for lie-truth discrimination. Legal criteria have been variously set up across different jurisdictions, aiming to provide triers of fact with standards to evaluate witness truthfulness and decide on witness credibility. Most importantly, witness “demeanor” is the crucial aspect that judges and jurors are instructed to consider. This aspect does not refer to the content of evidence, but, as defined in the classical Goffman's studies, it concerns “*deportment, dress, bearing*” (7), and includes every visible and/or audible expression manifested by the witness in front of the Court or any interviewer, either fixed or variable, voluntary or involuntary, simple or complex (8).

Across different national contexts, guidelines and Court rulings have supplied specific instructions on how to evaluate a witness' demeanor. For instance, in the US, the 2017 Manual of Model Criminal Jury Instructions of the Ninth Circuit Jury Instructions Committee recommends jurors to consider some intrinsic features of witness testimonies, such as the witness' manner of testifying and the intrinsic reasonableness of witness reports. In Canada, a recent ruling (*Breed v. Breed, 2016, NSSC 42*) referred to specific aspects of testimonies, such as the consistency of external and internal reports (“*what are the inconsistencies and weaknesses in the witness' evidence, which include internal inconsistencies, prior inconsistent statements, inconsistencies between the witness' testimony, and the documentary evidence, and the testimony of other witnesses”*), accuracy and quantity of details (“*sufficient power of recollection to provide the court with an accurate account”*), and exposure modality (“*Was the evidence provided in a candid and straight forward manner, or was the witness evasive, strategic, hesitant, or biased”*). The consideration of internal and external consistency-of-witness accounts is among the 14 rules of thumb listed by Douglas in a paper presented at the 2004 Australian Institute of Judicial Administration Tribunal's Conference (9). In Europe, there are many examples of the criteria adopted by Courts in evaluating witness evidence. The judgement on *Berezovsky v Abramovich*, set up in UK in 2007, includes, among these criteria, confidence (“*witnesses can easily persuade themselves that their recollection of what happened is the correct one*,” p. 14), specificity of reported details (“*careful and thoughtful answers, which were focused on the specific issues about which he was being questioned*,” p. 18), and memory consistency (“*I found Mr. Berezovsky's evidence (and that of his witnesses) in relation to this issue to be vague, internally inconsistent*,” p. 23). In 1988, the Spanish Supreme Court held a sentence (Sentencia del TS, Sala de lo Penal, de 28 septiembre 1988, RJ 7070) focusing on external corroboration (“*verisimilitude*”) and over time consistency of witness accounts (“*persistence in incrimination*”) (10). The Italian Supreme Court (Cassazione) has underlined the importance of judges making a critical evaluation of a witness' evidence, expressly in cases of victims of sexual abuse, by paying special attention to consistency both across different interviews, and with other witnesses of the same crime. However, decision *no. 37988 of September 13*th*, 2016* leaves a “*large margin of appreciation regarding the methods for controlling witness credibility in specific cases*.” In sum, this brief juridical review shows that the legal evaluation of witness testimonies is generally based on a subjective evaluation by judges and jurors of the truthfulness on witness reports (11).

## Experiential Grounding of Legal Criteria

As surprising it may be, judges and jurors evaluate witness evidence based upon categories which correspond to what laypeople usually consider as indicators of truthful/deceptive behavior. In other words, the purity of legal judgement seems to be grounded in the subjective experience and commonsense of triers of fact. In the following section we will review a large body of evidence concerning the psychological processes underpinning the legal criteria for the assessment of truthfulness of witness accounts. Paralleling the jurisprudential review, we will enucleate a set of psychological categories related with lie/truth deception, which might be equated to the legal criteria above presented.

### Emotional Features and Expressive Indices

The so-called emotional approach to lie detection emphasizes that lying is much more arousing than merely telling the truth. When lying, individuals experience a range of internal states (physiological and psychological) associated with specific behavioral indices (12–14). As a forensic instance, during a police interview, a suspect trying to propose a false alibi might experience fear of being caught; shame or guilt might be associated with violation of moral standards implied by lying; finally, a liar might also experience excitement, satisfaction or happiness for getting away with lies (15). Individuals are thus forced to mask the associated physiological and behavioral reactions, so that deception detection can be based on an accurate analysis of these patterns escaping control. It is thus not surprising that, among the criteria recommended across jurisdictions to discriminate lies from truthful accounts, the legal systems consistently encourage an analysis of the witness “demeanor,” including all emotional manifestations implied in a witness testimony.

The pioneering work by Paul Ekman has emphasized the role of emotion identification in deception detection. Derived from the Darwinian evolutionary principles, Ekman's neurocultural theory of emotions considers the expression of emotions as a universal signaling system for organisms to communicate conspecifics in the presence of a predator or other critical cues for the survival of the individual and the species (15, 16). This signaling system includes physiological reactions and behavioral indices, many of which are conveyed by facial expressions. Culture teaches humans how to manage emotions in social contexts, by intensifying, de-intensifying or also dissimulating a given expressive pattern [i.e., display rules; (17)]. Emotion identification responds to the evolutionary need to ensure survival from danger, hence individuals learn to decode emotional signals from interactions with their conspecifics. Through daily life experiences, laypeople refine their capacity to identify others' behavioral manifestations and any form of emotional expression. Different theoretical accounts and empirical findings have emphasized the cultural variability of the production and perception of emotions (18, 19). Despite these different positions, on the whole, the emotion-based approach to deception can explain the reluctance of law enforcement and other professional lie-catchers to undergo extensive lie detection training (20): individuals generally rate themselves as sufficiently expert to correctly identify universal emotional signals; they also consider perceived indicators of deception based on non-verbal behavior as sufficiently accurate as actual indicators of deception (21). It follows that a professional lie-catcher adds his/her experience in lie identification to the competence attained from daily life experiences. However, as the above-mentioned studies by DePaulo et al. (2), and Bond and DePaulo (3) pointed out, the laypeople's ability to discriminate lies from truth based upon non-verbal signals is only slightly above chance.

### Cognitive Complexity

The cognitive approach to lie detection is based on the empirical observation that, during a face-to-face interview, lying is much more cognitively demanding than telling the truth (4, 22). Simulating an episodic event or a story requires access to executive control processes involved in suppressing the truth, searching for information in long-term memory, and packing a lie in working memory (23). More specifically, the liar is asked to perform several cognitive tasks consuming high resources: (1) to produce a lie that is plausible and coherent with what the listener knows or may find out, (2) to keep in mind his/her inventions to report consistent statements in the future, (3) to monitor his/her reactions not to look deceptive, as well as the listener's reactions to make sure the listener does not distrust him/her, and (4) to suppress the truth (24, 25).

The cognitive approach to lie detection supports a consideration of the intrinsic characteristics of verbal reports to discriminate lies from truthful accounts. Recent studies have underlined that this approach downplays the role of other cognitive processes intervening in deception and does not include an adequate consideration of individual differences (22, 23, 26). However, the brief jurisprudential review referred to above shows that judges and jurors are generally instructed to pay attention to internal and external consistency in witness narratives, associated sense of confidence, quantity, and specificity of reported details, and intrinsic reasonableness and plausibility of witness' accounts. Such aspects are usually considered genuine proxies of accuracy by the empirical literature on autobiographical memory in forensic settings.

To illustrate, Peace and Porter (27) compared the properties of genuine vs. fabricated memories of a traumatic experience, and showed that, over a 6-month period, genuine accounts were more consistent, detailed, rich of contextual, and emotional information, and rated as more plausible than fabricated narratives. However, liars might also be highly motivated to keep consistent reports to protect either themselves or somebody else from the unwanted consequences of legal proceedings. At odds with the beliefs of laypeople and law professionals, consistency might also be indicative of lying rather than truth telling, especially in cases of repeated assessment of a suspect (28, 29).

Moreover, the sense of confidence exhibited by the interviewee has been shown to have a powerful persuasive effect on jurors (30). Experimental studies have met judicial case studies concerning innocent people being accused, tried, convicted, imprisoned, and sometimes executed for crimes they did not commit, following the testimony of individuals high in self-confidence or interacting with highly confident co-witnesses (31, 32). The persuasive effect of confidence has, however, been found to be moderated by a number of factors, such as the extension of witness reports (33), format of questioning (34), role of the interviewer [i.e., prosecutor vs. defense attorney, (35)], information provided to jurors to enhance skepticism (36), and reliance on an expert witness (37).

Finally, the phenomenological richness of details of reports sustains the interviewer's feeling that the interviewee's mental representations exactly correspond to events which really occurred in the past, very different from events only imagined, beliefs or semantic knowledge (38–40). The former representations have been shown to display greater clarity, more visual details, and more details for smell, sound, taste, location, time, and setting than imagined events (41, 42). The extensive meta-analysis by Oberlader et al. (43) summarizes the results of 56 English- and German-language studies, including studies adopting Criteria-Based Content Analysis [CBCA; (44)]. The authors concluded that a content analysis of reports concerning really experienced events—such as sexual abuse and violent offenses—qualitatively differ from deceitful accounts. However, the use of content analysis tools on witness reports is problematic in that systems of categories do not have the same validity when applied to either children or adults [see (45, 46)], require specific training in clinical psychology and psychological assessment, and are not easy to handle by jurors and judges in legal proceedings (47).

### Paraverbal Aspects

Paraverbal cues are related with the emotional features of deceptive behaviors. Their investigation has been carried out not only in police and legal settings, but also in the workplace as a strategy for getting employment, advancements, or to avoid punishment (48, 49). Paraverbal behavior concerns the way the interviewee communicates his/her accounts during a face-to-face interaction, and, according to Sporer (23), reveals the interviewee's nervousness associated with fabricating a deceptive report.

In the above-mentioned meta-analysis by DePaulo et al. (2), only two of the whole set of paraverbal indicators considered by studies were found to be significantly and positively associated with deception, i.e., pitch and vocal tension, while taking time was found to be shorter in deceptive statements than in truthful ones. Given relevant differences in samples, methods, and construct operationalization across studies, results from that meta-analysis were rather contradictory. A new meta-analysis by Sporer and Schwandt (50) was run on a small subset of paraverbal behaviors, i.e., message duration, number of words, speech rate, response latency, unfilled pauses, filled pauses, speech errors, repetitions, and pitch. In this study, the authors also included a broad set of moderators, i.e., the interviewee's preparation, motivation, content of the deceptive message, sanctioning, degree of interaction between experimenter and participant and type of experimental design, and operationalizations used. In this study too, the pattern of effect sizes was rather heterogeneous: only pitch, response latency and speech errors positively related with deception, while message duration was negatively associated with deception. However, the results were significantly influenced by all moderators, indicating that the interviewee's individual characteristics largely influence the interviewer's ability to discriminate lies from truth based on paraverbal indices. Moreover, lie detection seems to be based on paraverbal behaviors especially in low familiarity situations, while individuals preferably rely on verbal indices when facing highly familiar situations (51). Finally, a very recent meta-analysis by Hauch et al. (1) on the effects of training interviewers on detection abilities reported a medium effect size on lie accuracy for verbal cues, while training on paraverbal behaviors, alone or in association with other non-verbal cues, only resulted in marginal effects.

## Aim and Hypotheses

The above-reviewed studies indicated that the individual's capacity to discriminate lies from truth is far from accurate and poorly related with the individual's confidence in his/her detection (6). As highlighted above, both law enforcement and non-professional interviewers base their evaluations of truthfulness on some experiential criteria which can be matched with categories largely investigated in psychological studies on lie and deception, i.e., emotional features, cognitive complexity, and paraverbal aspects of interviewees' reports. However, as noted above, research work on these issues has demonstrated that such criteria are, at the very least, questionable (1, 3, 28, 29, 32, 47, 51). Nevertheless, legal systems across different jurisdictions consistently recommend relying on them in assessing the truthfulness of a witness and his/her credibility. To our knowledge, no studies so far have attempted a systematic investigation of the psychological underpinnings of these criteria in a controlled context such as a lab setting. Furthermore, a quantifiable control on the judges' individual characteristics and expectations in supporting the accuracy and confidence of lie/truth detection is lacking in previous research work.

Following the above-reviewed studies, we designed an experimental study aimed at providing a better understanding of the criteria adopted in lie/truth detection, and to relate these criteria with accuracy and confidence in lie/truth identification. To this end, we adopted two perspectives of investigation in a mixed model design: we were indeed interested not only in the capacity of naïve people (including in this category Court judges, jurors, and professional interviewers) at lie/truth detection from a videotaped interview, but also in assessing the interviewee's detectability as a liar or truth-teller by our judges; additionally, we investigated the associations of these criteria with lie/truth detection not only from the judge's perspective, but also from the perspective of the interviewee. The combination of these two perspectives of investigation enabled us to control for the judge's dispositional preference toward one or more criteria.

In employing an experimental manipulation, we sought to improve control issues for our study, without decreasing the generalizability of our results [see the meta-analysis by Hartwig and Bond, (52), on the stability of lie detection across different contexts]. We thus administered to a sample of naïve “judges” a random sequence of videotaped interviews of liar vs. truth-teller “interviewees,” and we tested the hypothesis that the judges' ability at lie/truth detection will be moderately accurate (1–3), and poorly associated with the judges' confidence in their evaluation (6); furthermore, we predicted that accuracy for truth identification will exceed that of lie detection (3). We finally expected that judges would support lie detection through experiential criteria concerning emotional and expressive features, cognitive complexity, and paraverbal aspects conveyed by the interviewees (2, 15, 23, 50). We also explored the associations of these criteria with both the judge's ability at lie/truth detection and with the evaluation of deceitfulness/truthfulness assigned to each interview.

## Method

### Design

The study adopted a mixed model design with the videotaped Interview Condition (Liar vs. Truth-Teller) as a between-subjects (fixed effect) factor, and a random effect for a sample of judges evaluating interviewees' behavior. The dependent variables were: (a) detection accuracy, (b) confidence in detection accuracy, (c) and the experiential criteria adopted to support detection.

### Samples

The sample of judges consisted of 50 Italian volunteers (25 women), aged between 20 and 36 (*M* = 24.54; *SD* = 3.41), with an average level of education of 13.96 years (*SD* = 1.62). Each judge watched and listened to 50% of the videotaped interviews randomly selected from a pool of 20 interviews (see below), distributed across the two conditions (Liar vs. Truth-Teller, from 3 to 7 videos for each condition, 10 in total).

The sample of interviewees consisted of 20 Italian volunteers (10 women), aged between 21 and 28 (*M* = 23.50; *SD* = 1.91), with an average level of education of 13.75 years (*SD* = 1.33). Participants were matched for the two experimental interview conditions (10 Liar vs. 10 Truth-Teller). Each participant was administered an interview that was recorded to be subsequently shown to judges. Each videotaped interview was watched and listened to by 50% of the sample of judges.

All participants of both samples were recruited among students and experimenters' acquaintances. There was neither kinship, nor friendship, nor familiarity between the two samples: Each judge was unknown to each interviewee, and vice versa. Data were collected anonymously, and participants were preliminarily presented with an informed consent form. The Ethical Committee of the Department of Education, Psychology, Communication, University of Bari approved the study.

### Measures and Procedure

#### Videotaped Interviewees Sample

The 20 interviewees were invited to participate in an experiment on the cognitive processing involved in an interview. Two separate sessions (Writing session and Videotaped Interview session) were arranged, and participants were randomly assigned to one of two videotaped Interview Conditions (Liar vs. Truth-Teller).

#### Writing Session

All participants were previously contacted by email or telephone and invited to provide a brief written story. Specifically, in the Liar condition, participants were asked to fabricate a fake holiday that supposedly happened in the last 12–18 months (e.g., “*Lie to me about your last holiday. So, for example, if your last holiday was to Paris where you visited galleries, we ask you to make up a holiday to a place you have never been to - for example, Barcelona - and lie to us about what you did - for example, went out with friends and swam with dolphins -”)*. In the Truth-Teller condition, participants were asked to describe a holiday they really had in the last 12–18 months. Participants were invited to send us the texts by email, in order to prepare a base for the subsequent interview session.

#### Videotaped Interview Session

The day after the writing session, each participant was asked to sit relaxed in the lab and to talk facing a camera placed in front of him/her. Participants were informed that they were to talk about the holiday they had written of the day before, by talking to the video camera. It was also specified that the experimenter who performed the video interview did not know if the holiday they were recounting was real or simulated. As a consequence, participants were asked to be as credible as possible. This session was conducted by an experimenter who was not involved in the previous phase, and was unknown to participants. It consisted of two phases:

1. *Free telling phase*. Participants were asked to just relax and sit in front of a camera and to talk freely about the holiday they wrote of the day before (Liar vs. Truth-Teller condition), for about one minute and a half. They were asked to describe their trip, without worrying about timing; and were stopped when they had talked for long enough.
2. *Questions phase*. Each participant was interviewed in order to specify some details which had already been provided in the story or to give additional contextual details (weather, delay, scheduled event). For example, if an interviewee had said that he/she had a 1-week trip on Paris with his/her parents, he/she would be asked: “*Could you please tell me about some of the neighborhoods you visited?”* or “*How was the weather the first day you arrived in Paris?”* This phase lasted for about one and a half minutes.

Once both sessions were completed (about 3 min in total), participants were debriefed and thanked.

#### Judges' Sample

The sample of 50 judges was recruited by being asked whether they were willing to participate in an experimental study on evaluating a videotaped interview. Each judge was tested in a unique session, sitting in a quiet room, and requested to watch on a computer screen a random sequence of 10 videotaped interviews taken from the whole pool and distributed across the two conditions of the design (Liar vs. Truth-Teller, from 3 to 7 videos for each condition, 10 in total). We employed an unequal number of truthful and lying videotaped interviews to avoid the judges' expectation that half of the interview would be lies.

The judges were then asked: (a) to detect to which interview condition the interviewee was assigned (Liar vs. Truth-Teller), (b) to evaluate the level of confidence in their detection on an 11-point scale (Confidence score, 0 = “not at all”; 10 = “very much”), and (c) indicate the criteria they adopted to support detection through answering an open-ended question.

#### Coding System for Criteria of Lie/Truth Detection

Each judge's answer concerning the criteria adopted to support detection was transcribed verbatim and a coding system was applied, based on the psychological categories presented in the Intro. The authors identified four main categories of criteria, comparable with the psychological constructs presented above. The first category includes general *emotional features* of the interview, i.e., the judge emphasized the interviewee's ability to emotionally involve the viewer, the interviewee's calmness vs. nervousness, the coherence between story content and emotions expressed, the coherence between behavioral indices and emotions expressed. The second category includes the judges' mentions of specific *expressive indices*, such as the interviewee's direction of the gaze; mimic, and facial expressions (smiles, stillness, lip movements); body gestures (touching your nose, scratching elbow, etc.); and physical characteristics of the interviewee (appearance, bodily attitudes). The third category refers to the *cognitive complexity* of the story, i.e., the judge stated whether the interviewee's account appeared consistent, truthful, detailed, and vivid. The last category refers to the *paraverbal aspects* of the report, i.e., the judges explicitly mentioned the exposure clarity, fluency of the speech vs. hesitation, reactivity and/or readiness of response, latency times, confidence, and/or spontaneity in the exhibition, voice tone, participation vs. acting, and linear vs. fragmented exposition. One point was assigned for each criterion mentioned. Two trained coders—who were blind to each other's results—independently scored half of the total 500 judges accounts (50 judges × 10 videotaped interviews). The interrater reliability was high for such a scoring (*r*_Emotional features_ = 0.94; *r*_Expressive indices_ = 0.96; *r*_Cognitive complexity_ = 0.90; *r*_Paraverbal aspects_ = 0.90). (See Appendix for an example of the coding system).

## Results

### Judges' Level

#### Descriptive and Correlational Analyses

For each judge, we analyzed his/her lie/truth detection capacity and the criteria adopted to support detection. To this end, we computed the following indices: (a) Detection Accuracy was obtained by averaging the accuracy scores for each observed interview in the two conditions of the design (0 = “error”; 1 = “correct”; range 0–1 for liar and truth-teller conditions, respectively); (b) Detection Confidence was obtained by averaging the Confidence scores for each watched interview separately for the two conditions of the design (range 0–10 for both liar and truth-teller conditions); (c) frequencies of occurrence of each category of experiential criteria were transformed into proportions; for each condition of the design (Liar vs. Truth-teller) we computed the total occurrence of each category across all videotaped interviews shown to the judge, and divided that sum by the maximum occurrence of categories for that judge. This computation takes into account the individual's distribution of category occurrences, normalizing for the individual's propensity to prolixity. Table 1 showed descriptive analyses for the judges' level. Overall, results showed that judges report a medium level of Accuracy and a medium-high level of Confidence in detecting liar vs. truth-teller interviewees, and a low occurrence for the categories of experiential criteria, with Cognitive complexity and Paraverbal aspects as the highest experiential criteria mentioned to support detection. The parametric paired *t*-test revealed a significant effect of condition (Liar vs. Truth-teller) on the measure of Detection Accuracy, in that it seemed to be slightly easier for our judges to accurately detect truthful rather than deceitful interviews. Additionally, the *t*-test showed a significantly higher occurrence of Expressive indices in the evaluation of liars than truth-teller interviewees. The significant effect on the index of Detection Accuracy was further explored to evaluate if it was due to a different base rate of truthful videos presented to our judges as compared with lying ones. To this end, the entire sample of judges was divided into three subsamples viewing, respectively 3–4 vs. 5 vs. 6–7 truthful videotaped interviews, and separate *t*-test analyses were run on the measure of Detection Accuracy for each of the three subsamples. The effect of condition vanished when the base rate of truthful interviews was ≤ 50% (*t*s < |1.80|, *n.s*.), but it remains significant for the subgroup of judges viewing 6–7 truthful videotaped interviews [*t*_(14)_ = −3.01, *p* < 0.01].

**Table 1:** Descriptive statistics for judges' sample level (*N* = 50).

**Indices**	**Total interviews**	**Liar interview condition**	**Truth-Teller interview condition**	**Paired samples *t*-test (df = 49) [mean difference 95% CI]**
	**M (SD)**	**M (SD)**	**M (SD)**
Detection accuracy	0.53 (0.15)	0.46 (0.21)	0.60 (0.24)	−3.01^**^ [−0.23, −0.05]
Detection confidence	6.95 (1.09)	6.93 (1.23)	6.90 (1.17)	0.21 [−0.23, 0.29]
Emotional features	0.17 (0.10)	0.15 (0.11)	0.19 (0.13)	−1.83 [−0.08, 0.003]
Expressive indices	0.19 (0.10)	0.21 (0.11)	0.17 (0.14)	2.02^*^ [.00, 0.09]
Cognitive complexity	0.36 (0.15)	0.36 (0.22)	0.37 (0.18)	−0.43[−0.08, 0.05]
Paraverbal aspects	0.28 (0.13)	0.29 (0.17)	0.28 (0.15)	0.33 [−0.04, 0.06]

* p < 0.05;

** *p < 0.01*.

**Figure 1:**
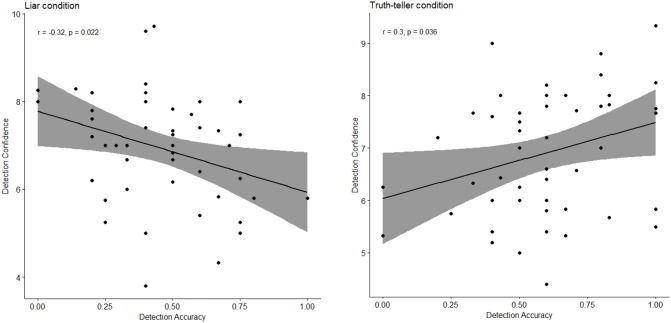
Scatterplot of correlations Detection Accuracy-Detection Confidence measures for judges' sample level (*N* = 50), for the liar **(Left)**, and truth-teller conditions **(Right)**.

Table 2 shows Pearson's zero-order correlations of all the indices described above for the judges' sample. Interestingly, for the liar condition, Detection Accuracy was negatively associated with Detection Confidence, whilst for the truth-teller condition the two indices were positively associated (see also Figure 1). Additionally, Confidence scores for the two conditions were strongly positively associated. Detection Accuracy in the liar condition was positively related to the occurrence of Emotional features of the reported story. By contrast, Detection Accuracy in the truth-teller condition was positively associated with the Cognitive complexity category and negatively associated with Expressive indices.

**Table 2:** Pearson's correlations for judges' sample level (*N* = 50).

**Indices**	**Detection accuracy liar**	**Detection accuracy truth-teller**	**Detection confidence liar**	**Detection confidence truth-teller**
Detection accuracy—truth-teller	0.00		
Detection confidence—liar	−0.32^*^		
Detection confidence–truth-teller		0.30^*^	0.71^**^
Emotional features—liar	0.30^*^		−0.33^*^
Emotional features—truth-teller		−0.04		0.12
Expressive indices—liar	0.09		0.05
Expressive indices—truth-teller		−0.35^*^		−0.11
Cognitve complexity—liar	−0.22		0.08
Cognitive complexity—truth-teller		0.43^**^		0.14
Paraverbal aspects—liar	0.02		0.07
Paraverbal aspects—truth		−0.16		−0.18

* p < 0.05;

** *p < 0.01*.

### Interviewees' Level

#### Descriptive and Correlational Analyses

This set of analyses reverses the logics of those described in the previous section, since our aim in designing the study was also to assess the interviewee's detectability as either lying or truthful by our judges. For each videotaped interview, we computed the following indices: (a) Detection Accuracy score was obtained by averaging the accuracy scores across judges (0 = “error”; 1 = “correct”; range 0-1); (b) Detection Confidence was obtained by averaging the Confidence scores across judges (range 0–10); (c) frequencies of occurrence of each category of experiential criteria were transformed into proportions; we computed the total occurrence of each category across judges, and divided that sum by the maximum occurrence of categories mentioned by all judges for that interview. Table 3 showed descriptive analyses for the interviewees' level. Overall, these results substantially confirmed those obtained for the judges' level. Medium levels of Accuracy and medium-high levels of Confidence were observed for the detection of both liar and truth-teller interviewees. Additionally, a low occurrence of the categories of experiential criteria was found to be associated with detection, with Cognitive complexity and Paraverbal aspects as the highest criteria mentioned. An independent samples *t*-test was run on all indices considered in the study (Detection Accuracy, Confidence, and experiential criteria), with the design condition (Liar vs. Truth-teller) as a between-subjects factor. Given the limited sample size (*n* = 10 videotaped interviews for condition), the non-parametric bootstrapping method was used as a robust estimation of *t*-test. Bootstrapping provided a confidence interval (CI) around the mean difference, which is significant if the interval between the upper limit (UL) and lower limit (LL) of a bootstrapped 95% CI does not contain zero, which means that the difference between the two groups is different from zero. Albeit not significant (CI includes 0), Detection Accuracy appeared to be slightly higher in the truth-teller condition than in the liar condition, confirming the findings for the judges' level. Furthermore, a significantly higher occurrence of Emotional features was observed in the evaluation of truth-teller interviewees. Bootstrapped Pearson's zero-order correlations were run among indices on the total sample of interviewees, and separately for the two interview conditions (Liar vs. Truth-teller; Table 4). For the interviewee's level, the association between Detection Accuracy and Confidence, albeit non-significant, is consistent with the judges' level, i.e., negative for the liar condition and positive for the truth-teller condition. However, none of the associations among the categories was found to reach the significance level.

**Table 3:** Descriptive statistics for total sample of interviewees and for the two interview conditions (*N* = 20; 1,000 Bootstrapped Samples).

**Indices**	**Total sample (*N* = 20)**	**Liar condition (*n* = 10)**	**Truth-Teller condition (*n* = 10)**	**Independent samples *t*-test (df = 18) [mean difference 95% CI]**
	**M (SD)**	**M (SD)**	**M (SD)**
Detection accuracy	0.53 (0.19)	0.46 (0.21)	0.61 (0.15)	−1.83 [−0.30, 0.01]
Detection confidence	6.95 (0.41)	6.99 (0.53)	6.92 (0.25)	0.41 [−0.26, 0.46]
Emotional features	0.17 (0.05)	0.15 (0.05)	0.20 (0.05)	−2.14^*^ [−0.09, −0.00]
Expressive indices	0.20 (0.06)	0.22 (0.07)	0.18 (0.05)	1.46 [−0.01, 0.09]
Cognitive complexity	0.34 (0.06)	0.33 (0.06)	0.35 (0.06)	−0.80 [−0.07, 0.03]
Paraverbal aspects	0.28 (0.05)	0.29 (0.06)	0.27 (0.04)	1.28 [−0.02, 0.07]

* *p < 0.05*.

**Table 4:** Pearson's correlations for total sample of interviewees and for the two interview conditions (*N* = 20; 1,000 Bootstrapped Samples).

**Indices**	**Total sample (*****N*** **=** **20)**		**Liar condition (*****n*** **=** **10)**		**Truth-Teller condition (*****n*** **=** **10)**	
	**Detection accuracy**	**Detection confidence**	**Detection accuracy**	**Detection confidence**	**Detection accuracy**	**Detection confidence**
Detection confidence	−0.21 [−0.65, 0.50]		−0.34 [−0.79, 0.53]		0.25 [−0.26, 0.77]
Emotional features	−0.06 [−0.36, 0.35]	0.16 [−0.25, 0.60]	−0.39 [−0.81, 0.28]	0.20 [−0.48, 0.72]	−0.13 [−0.72, 0.69]	0.35 [−0.20, 0.84]
Expressive indices	−0.16 [−0.50, 0.22]	0.01 [−0.63, 0.52]	0.08 [−0.40, 0.59]	0.14 [−0.58, 0.76]	−0.29 [−0.74, 0.27]	−0.56 [−0.87, 0.05]
Cognitive complexity	−0.02 [−0.40, 0.41]	0.18 [−0.23, 0.54]	−0.32 [−0.83, 0.38]	0.29 [−0.44, 0.82]	0.18 [−0.31, 0.77]	0.05 [−0.54, 0.60]
Paraverbal aspects	0.27 [−0.19, 0.62]	−0.37 [−0.72, 0.13]	0.52 [−0.20, 0.85]	−0.58 [−0.89, 0.05]	0.23 [−0.34, 0.69]	0.18 [−0.78, 0.79]

#### Receiver-Operating-Characteristic (ROC) Analysis

Generally speaking, a receiver-operating-characteristic (ROC) analysis (53) is used to determine the diagnostic performance of a test to discriminate diseased cases from normal cases (i.e., diagnostic accuracy). The accuracy of the test depends on how well the test separates the two categories or conditions (diseased vs. normal). Analogously, in our data, we adopted the ROC analysis to determine the accuracy of judges' detection (the above-referred as test) in discriminating truthful from deceitful videotaped interviews (the above-referred as diseased vs. normal cases). Our measure on which diagnostic accuracy was tested corresponds to the judges' raw detection whether the interviewee belongs to either a liar condition or a truth-teller condition, regardless if the detection was correct or not. This measure was obtained by summing up all detection scores provided by the 25 judges for each videotaped interview (score 0 = liar's detection, score 1 = truth-teller's detection, regardless of the correctness of such a detection). This aggregate Raw Detection index ranged from 0 to 25, with higher values indicating a prevalence of truth-tellers' detection, and lower values indicating a prevalence of liars' detection.

The Raw Detection index was employed as a measure indicating the diagnostic power that an interviewee falls into one condition (1 = truth-teller) or the other (0 = liar). In our data, the truth-teller condition was employed as a state variable indicating the “true category” to which the interviewee belongs. The value of the state variable indicates which category should be considered positive (in our case 1 = truth-teller). Higher values indicate a greater probability of positive category (1 = truth-teller).

Diagnostic accuracy is measured by the Area Under the Curve (AUC), which takes values from 1 (perfectly accurate discrimination) to 0 (perfectly inaccurate discrimination). In general, an AUC of 0.5 suggests no discrimination, from 0.7 to 0.8 is considered acceptable, from 0.8 to 0.9 is considered excellent, and more than 0.9 is considered outstanding (54). The ROC analysis ran on the present data yielded an AUC of 0.61, indicating a little above typical power to discriminate truthful from deceitful videotaped interviews.

### Multilevel Analyses

The following analyses were run to control for the judges' individual variability in accuracy, confidence, and experiential criteria for lie/truth detection. To this end, we tested a random intercept model with the package *lme4* (55) for multilevel analysis through R (56). This analysis was separately applied to the indices of Detection Accuracy and Detection Confidence, and to the proportion of occurrence of the four categories of criteria for each interview shown to each judge (Emotional features, Expressive indices, Cognitive complexity, and Paraverbal aspects). Only for the Detection Accuracy index, given the dichotomous nature of the dependent variable (0 = “error”; 1 = “correct”), the estimated model was a logistic regression.

As a general procedure, we first estimated a model with fixed factors only and we included the Interview condition (Liar vs. Truth-Teller) as a fixed effect variable. Along with this factor of the design, we also intended to control for the congruence between demographic characteristics of judges and interviewees: given that judges and interviewees do not differ as to their age [*t*_(68)_ = 1.28, *n.s*.], we only considered gender congruence as an additional fixed factor in our models (dichotomous indicator, 0 = non-congruent; 1 = congruent). We finally included the individual variability of judges evaluating interviewees' behavior as a random factor (judges' ID). An a priori power analysis was applied through the *lme4, simglm* (57), and *paramtest* (58) R packages, on a model with two fixed dichotomic factors, and a random factor with σ^2^ = 0.50. With a medium effect size = 0.50 for the two fixed factors, *p* < 0.05, a total of 500 observations (50 judges × 10 interviewees), and simulated samples = 100, the analysis yields a power >0.75.

The fit of our models was estimated by applying the *car* R package to obtain the Wald test statistics (59). The AIC and BIC indices were computed to enable a comparison between the model with only fixed effects (Interview condition and gender congruence) with the model with both fixed and random effects (judges' ID). As Table 5 shows, the only significant fixed effect was found for the Interview condition on the measure of Detection Accuracy, in that truth-teller interviewees were more accurately identified than liars (β = 0.62, z = 3.38, *p* < 0.001; Wald test = 13.02, *p* < 0.001; AIC = 684.31; BIC = 696.96). For none of the dependent variables entered in the model the effect of gender congruence judge-interviewee was found to be significant. Furthermore, for none of our dependent variables, controlling for the judges' individual variability resulted in a significant improvement of the model [AIC and BIC were lower for the model with fixed effects only; (60)]. Finally, in order to rule out any confounding due to the interviewees' variability, the multilevel models were also run by including a random intercept for interviewees' ID. The last columns of Table 5 display the fit indices for the models with both fixed effects (Interview condition and gender congruence) and interviewees' variability as random factor. As shown in the table, the inclusion of the random factor does not improve the fit of the models (see AIC and BIC indices in the last column of Table 5), thus confirming the stability of our results also after controlling for interviewees' variability.

**Table 5:** Multilevel analysis on the measures of the study, testing the effect of interview condition, and gender congruence (fixed effects) and judges' and interviewees' individual variability (random intercept models).

	**Fixed effects only**					**Fixed and random effects (Judges' ID)**			**Fixed and random effects (Interviewees' ID)**		
	**Interview condition (Liar vs. Truth-teller)**	**Gender congruence judge-interviewee**	**Wald test (df =1)**	**AIC**	**BIC**	**Wald test (df = 1)**	**AIC**	**BIC**	**Wald test (df = 1)**	**AIC**	**BIC**
Detection accuracy	0.62 (*z* = 3.38^***^)	−0.22 (*z* = −1.21)	13.02^***^	684.31	696.96	12.87^***^	686.31	703.17	4.92^*^	667.13	683.99
Detection confidence	−0.08 (*t* = −0.42)	0.01 (*t* = 0.05)	0.18	2125.86	2142.71	0.07	2080.31	2101.38	0.17	2133.96	2155.03
Emotional features	0.05 (*t* = 1.83)	0.00 (*t* = 0.13)	3.38	131.65	148.51	3.38	150.26	171.33	2.18	149.66	170.73
Expressive indices	−0.03 (*t* = −1.47)	0.03 (*t* = 1.16)	3.52	12.27	29.13	3.76	27.87	48.94	1.71	30.80	51.87
Cognitive complexity	0.01 (*t* = 0.24)	−0.02 (*t* = −0.55)	0.36	453.34	470.20	0.35	451.64	472.71	0.33	471.37	492.44
Paraverbal aspects	−0.02 (*t* = −0.59)	−0.01 (*t* = −0.21)	0.39	319.48	336.34	0.33	330.05	351.12	0.27	330.05	351.12

* *p < 0.05*,

*** *p < 0.001*.

*AIC, Akaike's Information Criterion; BIC, Bayesian Information Criterion*.

*The lme4 package returns z-tests (logistic model) and t-tests for fixed effects, and estimated variance for random effects; the Wald test for the models has a chi-square distribution*.

In sum, truth detection appeared to be slightly easier than lie detection, regardless of the peculiar individual characteristics of the judge evaluating the interviewee's behavior. Furthermore, the associated sense of confidence in detection and—surprisingly—the adoption of the experiential criteria to support detection accuracy resulted as being completely unaffected by the individual's variability. Overall the present results are consistent with results reported in the preceding sections of the present paper, in spite of the different measurement models adopted (i.e., for Detection Accuracy, average measure across judges vs. dichotomous items in multilevel modeling).

## Discussion

In the current study, we aimed to assess the ability of naïve judges to discriminate deceitful vs. truthful reports by watching a videotaped interview (the judges' level) in a lab context, along with the interviewees' detectability as liars or truth-tellers from the judges (the interviewees' level). We also aimed to identify the criteria adopted by lay people to justify lie/truth detection and relate them with detection accuracy and confidence. To accomplish our goals we adopted a multilevel approach, requiring a sophisticated data-analytic methodology for an experimental design. Two sets of analyses were conducted to account for the structure of our data (judges and interviewees levels). Overall, results are consistent for the two levels of investigation: Lie/truth detection was found to be moderately accurate across judges and across interviewees, but a slightly higher accuracy was observed for detection of truthful accounts than deceitful ones; furthermore, judges appeared to be moderately confident that their detection was accurate.

The accuracy-confidence link showed an interesting pattern of results across the liar vs. truth-teller conditions: when naïve people are faced with a deceitful report, detection—although accurate—appears to be negatively associated with confidence; contrariwise, naïve people seem more confident when accurately identifying a truthful report. In other words, “I am not too sure when I detect a lie, even if it is really a lie.” It thus seems that detecting a lie has a greater “cost” in terms of confidence, for a kind of “conservative attitude” when people have to identify an unknown other as a liar. In that our results are consistent with DePaulo et al. conclusion, that judges are more confident when they are evaluating actual truths (accurate truth detection) as compared to when they are evaluating actual lies (accurate lie detection) (6).

As regards the criteria, results showed that the interviewee's physical characteristics, his/her mimic, and facial expressions, his/her gaze direction and body gestures were the indices most mentioned to detect a liar than a truth-teller interviewee. The interviewee's nervousness, and the incoherence between story content, behavioral indices, and emotions expressed were the criteria most frequently adopted to accurately detect a liar; consistency of the reports, richness of details, and vividness and poor expressive manifestations were the most recurrent criteria to accurately detect a truth-teller interviewee. Finally, as shown by the ROC analyses, the strength to which deceitful vs. truthful reports were discriminated from each other was modest. Jointly considered, our findings sustain the idea that people are not accurate in detecting the deception of unknown people (61), and that they occasionally support detection through experiential criteria concerning internal features of the witness accounts (2, 3).

Our study shows that individuals selectively choose either emotional or cognitive indices to identify lies vs. truthful interviews. A possible explanation for this asymmetry might be that individuals are naturally trained to detect emotional signals as cues of deception (15, 62), so that they justify their feeling that the interviewee is truthful by relying upon an evaluation of emotional signals. This explanation is in line with neuropsychological and neurobiological studies which have underlined the role of specific neural circuits in deception detection (63, 64). Among those circuits, the amygdala and the anterior cingulate cortex have been shown to be activated in social judgement tasks, when decoding of emotional signals is particularly relevant for interpersonal cooperation, communication, social business, and management, and for the ultimate goal of individuals' and species survival (65–67). When individuals are alerted by emotional signals that a speaker is lying, reliance upon those signals disrupts the usual cognitive processing of verbal messages (20). It follows that, while an accurate evaluation of truthful interviews is supported by a controlled analysis of cognitive features of verbal accounts, lie detection is preferentially anchored to decoding emotional indices. Our study reveals that naïve judges keep a sort of implicit knowledge of this differential processing of lies and truthful reports, and this knowledge is reflected in the legal criteria suggested across different jurisdictions to evaluate witness' truthfulness and decide on witness credibility.

An important strength of our study is that the experimental approach enabled a sizeable control on the judges' individual dispositions and expectations when deciding on witness truthfulness. Previous meta-analyses have shown that the judges' individual variability does not play a crucial role on detection accuracy (61). However, as underlined by Aamodt and Custer (68), there is a paucity of studies available to assess the relationship between the individual's characteristics and accuracy in detecting deception. On this issue, a surprising outcome of the multilevel analysis is that judges' individual variability did not in any way affect the adoption of each one of the categories of experiential criteria to support lie/truth detection. In another words, the final decision concerning whether to believe a witness or not does not display any regularity with regards to the judge's individual disposition/bias nor with regards to the similarity between judges and interviewees (operationalized as gender congruence in our study). Among our findings, the only relevant effect concerns detection being more accurate for truth-teller than liar interviewees, but the amount of variance attributable to the judge's individual tendencies is worthless as compared with the variance due to the interview condition. These findings might lead us to conclude that detecting lies is generally more difficult than identifying truth. However, a more in-depth exploration of this difference in detection of truthful video interviews as compared with lying ones showed that the effect remains significant only when the base rate of truthful interviews exceeds lying ones. These findings might be accounted for by the so-called “veracity effect,” which predicts that detection accuracy is a linear function of message veracity, so that the probability for a judge of giving an accurate truth identification increases as long as the proportion of honest messages increases (69). This effect depends on the fact that people have a kind of “truth bias” (3), so that they are more prone to believe to others since they consider them essentially truthful (70). This bias is even underestimated in experimental settings as compared with real life interactions, where individuals are naïvely prone to accept deceptive messages as truth.

The present results have noteworthy implications in the forensic domain. To illustrate, gender congruence between jurors and witnesses can be an influential factor with respect to the composition of juries, especially in crimes such as rape or sexual aggression, where the victim and the defendant are the only people present on the crime scenario (71–75). Following these findings, we introduced gender congruence in our multilevel model to explore its role in predicting accuracy, confidence, and experiential criteria adopted by judges for lie/truth detection. However, our results prove that this factor is ineffective in lie/truth discrimination, hence could be neglected if the main task required of jurors were lie/truth detection. It should however be considered that our conclusions are based on an experimental paradigm in which naïve judges are required to decide whether an interviewee is truthful/liar when narrating a holiday narrative. This artificial setting cannot fully emulate the emotional and cognitive requirements of a sex crime trial. Another important point regards the role of judges with respect to a witness whose truthfulness has to be assessed. In the Italian legal system, as in other countries in which the witness undergoes classical cross-examination, the role of the judge and jurors is—except in specific instances—that of passive observers while attorneys and prosecutors run the witness' interview directly interacting with him/her. However, it is up to judges and/or jurors to draw conclusions on the witness' truthfulness, and credibility. The experimental setting adopted in our study, through the administration of a videotaped interview to a sample of naïve judges, attempts to emulate as far as possible the real context of a criminal proceeding, in which the interaction between judges and interviewees is generally precluded. Judges are thus forced to only focus on the intrinsic qualities of interviews and base on them lie/truth detection.

Findings from the present study highlight the experiential grounding of the legal criteria identified across jurisdictions to support the legal decision on witness credibility. The content analysis run on the answers provided by judges to the open-ended question yields a category system including references to emotional and expressive features of the interviewee's accounts, indices of cognitive complexity of reports, and paraverbal aspects concerning the story-telling regulation by the interviewee (2, 15, 23, 50). Each of these categories captures some features of the general concept of witness “demeanor” (7, 8), which triers of fact are requested to consider. The mention of these categories in the judges' responses was consistently assessed in our study, accounting for an experiential base for the jurisprudential criteria recommended across different national contexts. However, as the review in the introductory sections of the present paper shows, these criteria are largely disputed across scientific studies. People rate themselves as sufficiently expert at identifying lies from the interlocutor's physiological pattern and expressive behavior, but the laypeople's ability at lie/truth discrimination based upon non-verbal signals has been demonstrated as being only slightly above chance (2, 3). Narrative proxies of accuracy are controversial across studies, in that consistency, confidence, and phenomenological richness might also characterize deceitful and/or only imagined accounts (29, 32, 76, 77). In sum, the present findings confirm once more that, despite triers of fact struggling to apply jurisprudential principles and professional guidelines, the basis for the legal evaluation of witness evidence across jurisdictions is experiential and, as such, mainly unwarranted.

The results of the current study should be considered in the light of limitations and future perspectives. First, the composition of our two samples reduces the chance of massively generalizing our findings to a real lie/truth detection context: Our sample of judges did not include individuals belonging to categories especially concerned with witness' assessment (e.g., professional judges, jurors, police detectives, federal law enforcement, investigators, etc…), and interviewees' calmness and quietness when sitting in a “sterile” lab environment do not fully reproduce the real emotional state of a witness testifying in a criminal proceeding. Furthermore, the age and educational range both judges and interviewees is quite limited and this might compromise the generalizability of our findings. To illustrate, it has been shown that the ability to detect lie through visual information conveyed by facial expressions is attenuated in elderly as compared with young people (78, 79). It follows that our approach needs to be replied on samples of elder adults, which can be very often involved in criminal trials as victims of maltreatments or financial exploitation, perpetrators of crimes as internet frauds and sexual abuses, or professional judges. Second, and related with the first point, while in our study we controlled for gender congruence between judges and interviewees, the age limitation of our sample prevented us from assessing a possible effect of age congruence: the issue of age matching needs to be carefully considered in future replications, since studies do not converge on it, either showing no age-matching effect (80) or a significant effect only for young people (79). Third, our participants were instructed to give their accounts for about one minute and a half, and this temporal limitation might have influenced their ability at deception detection. Fourth, our study did not enable a direct interaction between judges and the interviewees, and the manipulation through a videotaped session hardly resembles a realistic situation of a court hearing. However, the issue of generalization does not represent a serious flaw in the study: As the recent meta-analysis by Hartwig and Bond (52) concludes, lie detectability is substantially stable for multiple cues, including in those cues both lab sessions and forensic settings. Finally, we used a straightforward questioning during the interviews, and avoided using suggestive techniques or imposing additional cognitive load on our interviewees. As studies adopting the cognitive-based approach to lie detection have variously shown, liars might find it particularly difficult to deceive when asked to maintain eye contact with the interviewer, expose their version of facts in reverse order, or answer unexpected questions (25). Future studies should implement the procedure by adding further constraints to the interview session and subsequent assessment.

Despite the aforementioned limits, the current work has several strengths. First, the number of observations was high (*n* = 500, 50 judges × 10 interviews) and based on full audiovisual modality (face, body, and speech), enabling the judge to evaluate many behavioral manifestations as usually done in naturalistic settings. Second, through a two-level design, the study provides a strong experimental control on individual variability in lie/truth detection from both the perspective of the judge and the interviewees (81). Third, our study investigated levels of accuracy and confidence and the criteria adopted for the evaluation of both deceitfulness and truthfulness, and the results we have obtained, as outlined before, clearly demonstrate that the two processes do not match completely. Fourth, the current work took into account not only indices traditionally associated with lie detection such as accuracy and confidence, but also the explanations on which naïve judges generally base their lie/truth detection. The current findings, although not exhaustive, thus represent a meaningful step forward in understanding the experiential base for the legal criteria adopted by courts to decide on witnesses' credibility.

In sum, the present study provides a contribution to the field of investigation of lie/truth detection, by showing through a lab manipulation that judges' assessment of witness' truthfulness and credibility rests upon experiential criteria, respectively focusing on the emotional features of the liar's account and on the cognitive complexity and scarcity of expressive manifestations of the truth-teller's account. The legal decision concerning witness' credibility is ultimately grounded on the experiential evaluation of judges and jurors, and, as such, it might gain benefit from the informative value of scientific evidence.

## Author Contributions

AC and TL designed the experiment, analyzed the data, and edited the manuscript. FB, SG, and RR collected the data and scored the protocols. All authors listed have made substantial, direct and intellectual contributions to the work, and approved it for publication.

### Conflict of Interest Statement

The authors declare that the research was conducted in the absence of any commercial or financial relationships that could be construed as a potential conflict of interest.
