# Supplementary material for: Accuracy, Confidence, and Experiential Criteria for Lie Detection Through a Videotaped Interview
Source: Front Psychiatry. 2019 Jan 22;9:748. doi: 10.3389/fpsyt.2018.00748 (PMC6357939; doi:10.3389/fpsyt.2018.00748)
Supplement: Supplementary file 1 [file Table_1.DOC]

**Appendix A**

Examples of judge’s naive criteria underlying his/her detection.

| **Judge #15**  Judge’s Detection = Liar; Videotaped Interview Condition = True Teller | | | | | | | | | |
| --- | --- | --- | --- | --- | --- | --- | --- | --- | --- |
| *‘He speaks all on one note, with little emotional investment. I think he is telling a lie because he blurs the artwork name. He touches his arm and chin all the time.’* | | | | | | | | | |
| **Categories** | | **Score** | | | | | |  | |
| Emotional Features | | 1 | | | *‘Little emotional investment’* | | | | |
| Expressive Indices | | 1 | | | *‘He touches his arm and chin all the time’* | | | | |
| Cognitive Complexity | | 1 | | | *‘He blurs the artwork name’* | | | | |
| Paraverbal Aspects | | 1 | | | *‘He speaks all on the note’* | | | | |
| **Judge #48**  Judge’s Detection = Liar; Videotaped Interview Condition = True Teller | | | | | | | | | |
| *‘He takes tactical breaks to think, his speech lacks emotion. He is lying there and never blinks. All his attention and memory are focused on technical details.’* | | | | | | | | | |
| **Categories** | | | **Score** | | | | | |  |
| Emotional Features | | | 1 | | | *‘His speech lacks emotion’* | | | |
| Expressive Indices | | | 1 | | | *‘He is lying there and never blinks’* | | | |
| Cognitive Complexity | | | 1 | | | *‘All his attention and memory are focused on technical details’* | | | |
| Paraverbal Aspects | | | 1 | | | *‘He takes tactical breaks to think* | | | |
| **Judge #48**  Judge’s Detection = Liar; Videotaped Interview Condition = Liar | | | | | | | | | |
| *‘She overemphasizes her speech, she is too much descriptive and too little emotional. She hardly ever blinks. She doesn’t know what she is talking about, getting redundant on non-emotional aspects (weather, landscape…). She is barely emotional invested, having a hard time remembering cities besides Florence.’* | | | | | | | | | |
| **Categories** | **Score** | | | | | |  | | |
| Emotional Features | 2 | | | *‘Too little emotional’; ‘She is barely emotional invested’* | | | | | |
| Expressive Indices | 1 | | | *‘She hardly ever blinks’* | | | | | |
| Cognitive Complexity | 2 | | | *‘She is too much descriptive’; ‘She doesn’t know what she is talking about, getting redundant on non-emotional aspects (weather, landscape...)’* | | | | | |
| Paraverbal Aspects | 2 | | | *‘She overemphasizes her speech’; ‘having a hard time remembering cities besides Florence’* | | | | | |
| **Judge #1**  Judge’s Detection = Liar; Videotaped Interview Condition = Liar | | | | | | | | | |
| *‘He touches his nose two times in a stereotipically way. It looks like he is well grounded about places but he had never been really there. He is dismissive and emotionally dull talking about the events. Apparently, he is nervous before the recall, too.’* | | | | | | | | | |
| **Categories** | **Score** | | | | | |  | | |
| *Emotional Features* | 1 | | | *‘He is nervous before the recall’* | | | | | |
| *Expressive Indices* | 1 | | | *‘He touches his nose two times in a stereotypically way’* | | | | | |
| *Cognitive Complexity* | 2 | | | *It looks like he is well grounded about places; ‘He is dismissive and emotionally dull talking about the events’* | | | | | |
| *Paraverbal Aspects* | 1 | | | *‘He had never been really there’* | | | | | |

The answers were translated into English only for the purpose of publication
